# Taxonomic recognition of some species-level lineages circumscribed in nominal *Rhizoplaca subdiscrepans* s. lat. (Lecanoraceae, Ascomycota)

**DOI:** 10.7717/peerj.9555

**Published:** 2020-08-03

**Authors:** Katarzyna Szczepańska, Jacek Urbaniak, Lucyna Śliwa

**Affiliations:** 1 Department of Botany and Plant Ecology, Wroclaw University of Environmental and Life Sciences, Wrocław, Poland; 2 Department of Lichenology, W. Szafer Institute of Botany, Polish Academy of Sciences, Kraków, Poland

**Keywords:** Lichenized fungi, Rhizoplaca, Cryptic species, Phylogeny, Taxonomy, Geographical distribution, Biodiversity

## Abstract

**Background:**

*Rhizoplaca subdiscrepans* (Nyl.) R. Sant., a saxicolous, placodioid lichen, is considered to have a worldwide distribution in warm-temperate to boreal-arctic areas in Asia, Europe and North America. However, recent studies have revealed that this species includes five unrecognized species-level lineages—‘subd A, B, C, D and E’. During research focused on the diversity of saxicolous lichens in mountainous areas of southern Poland, some interesting representatives of the genus *Rhizoplaca* were found. The main aim of our study was to determine the taxonomic status of the collected specimens by means of molecular tools and a comparative analysis of similar herbarium materials.

**Methods:**

Detailed morphological, anatomical and chemical examinations of reference material from Asia, Europe and North and South America focused primarily on a selected group of lecanoroid taxa with a placodioid thallus. In addition, 21 new generated sequences representing *Lecanora pseudomellea, Protoparmeliopsis muralis, Rhizoplaca opiniconensis, R. subdiscrepans* s. lat*.* and* R. phaedrophthalma* were selected for molecular study using the internal transcribed spacer region (ITS rDNA), together with 95 available GenBank sequences mainly from the genus *Rhizoplaca.*

**Results:**

Polish specimens that clustered with members of a potential species-level lineage ‘subd E’ of *Rhizoplaca subdiscrepans* complex were recovered. Comprehensive analyses of the lichen group led us to the conclusion that lineage ‘subd E’ represents *R. subdiscrepans* s. str. and that the taxon appears to have a limited geographical distribution and specific habitat preferences. Furthermore, some of the recently defined species candidates within *R. subdiscrepans* s. lat.—‘subd D’ and ‘subd A’—should be assigned to two previously known species of *Rhizoplaca*, namely *R. opiniconensis* (Brodo) Leavitt, Zhao Xin & Lumbsch and *R. phaedrophthalma* (Poelt) Leavitt, Zhao Xin & Lumbsch, respectively. These two species are characterized by phenotypic features observed as well in analyzed specimens representing lineages ’subd D’ and ’subd A’. Moreover, the representatives of these lineages demonstrate some differences in occupied habitat and geographical range that also correspond with the indicated species. Additionally, it was found that *Lecanora pseudomellea* B.D. Ryan is a strongly supported monophyletic lineage within *Rhizoplaca,* and therefore an appropriate new combination for the species is proposed.

## Introduction

The genus *Rhizoplaca* Zopf belongs to a large family of lichenized fungi, Lecanoraceae. It was first segregated out of the genus *Squamaria* DC. by Zopf (1905), and recognized later by Choisy (1929), who classified it under a new name *Omphalodina* M. Choisy. At the same time, *Omphalodina chrysoleuca* [*Rhizoplaca chrysoleuca* (Sm.) Zopf] was selected as the type species of the new genus. Later *Omphalodina* was included in the genus *Lecanora* as a section within the subgenus *Placodium* (Poelt, 1958); however, Leuckert, Poelt & Hahnel (1977) proposed this taxon at a generic level once more under its older name *Rhizoplaca*, on the basis of the following distinguishing features: umbilicate thalli, well-developed upper cortex, thick lower cortex, loose medulla and cupulate hypothecium.

In subsequent molecular studies, *Rhizoplaca* was found to be a highly polyphyletic genus diffuse within different clades among placodioid *Lecanora* spp. (Arup & Grube, 2000; Cansaran et al., 2006). Moreover, a cryptic species-level diversity was identified within seemingly morphologically homogeneous groups, such as *Rhizoplaca chrysoleuca* s. lat. (Zhou et al., 2006; Leavitt et al., 2016), *R. melanophthalma* s. lat. (Leavitt et al., 2011; Leavitt et al., 2013a) and *R. macleanii* s. lat. (Pérez-Ortega et al., 2012) as well as *R. subdiscrepans* s. lat. (Leavitt et al., 2016). It turned out that some of the indicated lineages were, in fact, good species candidates; these were described by Leavitt et al. (2013a) as *Rhizoplaca occulta*, *R. parilis*, *R. polymorpha*, *R. porterii* and *R. shushanii*. Some additional species were transferred into the genus *Rhizoplaca* by Zhao et al. (2016), e.g., *Lecanora nigromarginata*, *L. novomexicana*, *L. opiniconensis, L. phaedrophthalma* and *L*. *weberi*, while others were excluded from the genus, e.g., *Rhizoplaca spidophora* and *R. peltata*.

Currently, the genus *Rhizoplaca* comprises *c*. 24 species of lichenized fungi representing umbilicate to placodioid growth forms (Zhao et al., 2016). The thalli predominantly produce usnic acid in addition to various other substances. *Rhizoplaca* species occupy siliceous or weakly to moderately calcareous rock or, rarely, soil, and grow mainly in open, windy, cool and dry areas (Ryan & Nash, 1997). Species occur in the northern hemisphere (with their centre of diversity in Central Asia and western North America), as well as in South America and Antarctica (Leavitt et al., 2016; Zhao et al., 2016). Some of the known species show broad ecological preferences and geographical ranges, e.g., *R. chrysoleuca, R. melanophthalma* and *R. subdiscrepans,* whereas others have more restricted habitat requirements and limited distributions, e.g., *R. macleanii, R. maheui* and *R. marginalis* (Leavitt et al., 2016).

The subject of our study is *Rhizoplaca subdiscrepans* s. lat., a saxicolous lichen considered to be distributed worldwide, occurring in warm-temperate to boreal-arctic areas in Asia, Europe and North America (Ryan, 2001). In Europe it has been reported from Austria (Hafellner & Türk, 2001), the Czech Republic (Malíček, Palice & Vondrák, 2015), Italy (Nimis, 2016) and France (Roux, 2012), as well as Norway and Sweden (Santesson et al., 2004). It occurs on various siliceous rock types (basalt, rhyolite, granite and sandstone) and prefers exposed, warm and dry habitats (Ryan, 2001). The species was characterized by its yellowish-green, bullate-squamulose and polyphyllous thallus, with a pale brown lower surface and sessile apothecia possessing orange, pruinose discs. The thallus of *R. subdiscrepans* contains mainly usnic, placodiolic and pseudoplacodiolic acids (Arup & Grube, 2000) and rarely psoromic, lecanoric and norstictic acids (Ryan, 2001) as secondary metabolites.

Although the species was considered to be well-circumscribed, it recently proved to be polyphyletic and several unrecognized species-level lineages have been recovered in nominal *R. subdiscrepans* s. lat. by Leavitt et al. (2016). As a result of multigene analyses, these authors proposed five candidate species defined as *R. subdiscrepans* ‘subd A, B, C, D, E’, considered to be cryptic-species.

Our objective was to explain the taxonomic status of a putative representative of *R. subdiscrepans* (Nyl.) R. Sant. found during extensive field studies in the foothills foreland of the Sudety Mountains (Poland). Reference herbarium material from Asia, Europe and North and South America was examined, and sequences of gathered specimens were placed in a phylogenetic framework, including available GenBank sequences of placodioid lichens of selected species of the genera *Lecanora*, *Protoparmeliopsis* and *Rhizoplaca*. Based on morphological, chemical and molecular evidence, it was found that some of the recently defined species candidates within *R. subdiscrepans* s. lat. should be assigned to previously known species of the genus *Rhizoplaca*.

## Materials & Methods

### Taxon sampling

Material used in this study originated from the following herbaria: ASU, CANL (CMN), KRAM, M, MIN, PRA, WRSL and the private herbarium of K. Szczepańska (hb. Szczepańska). Our sampling focused on genera of lecanoroid lichens with placodioid thalli as follows: *Lecanora* (*L. pseudomellea*)*, Protoparmeliopsis* (*P. garovaglii, P. muralis*) and *Rhizoplaca* (*R. chrysoleuca, R. opiniconensis*, *R. phaedrophthalma, R. subdiscrepans*); holotypes of *Lecanora pseudomellea*, *Rhizoplaca opiniconensis* and *R. phaedrophthalma* were also analysed. Unfortunately, we were unable to study the original collection of *R. subdiscrepans* housed at the PC herbarium since type material at PC is unavailable for loan and we had no opportunity to visit the herbarium. All of the specimens listed in the text were included in the morphological, anatomical and chemical studies, but due to the age and poor condition of some samples, it was not possible to obtain sequences and include these in our phylogenetic analyses. New sequences generated for this study have been deposited in GenBank.

### Morphological and chemical study

All specimens were examined using light microscopy for the assessment of lobes, areoles, squamules and apothecia, especially the morphological characters mentioned in Leavitt et al. (2011) such as point of attachment (umbilicate/squamulose), thallus form (polyphyllous/monophyllous), lobe morphology (distinct/indistinct), appearance of upper surface (dull/glossy), upper surface colour (yellow-green/olive), apothecia morphology (sessile/constricted), apothecia pruinosity (pruinose/epruinose), ascospore shape (ellipsoid/subglobose) and ascospore size. Apothecial margin (persistent/excluded), height of hymenium, and size and shape of conidia (straight/arched) were also analysed.

For light microscopy, vertical, free-hand sections of apothecia were cut by a razor blade and mounted in water. Hymenium measurements were made in water and ascospore measurements in 10% KOH (K); the structure and coherency of paraphyses and the solubility of granules in the epihymenium were also tested with K. At least 10 measurements of morphological variables and measurements of 20 spores were made for each sample and their minimum and maximum values calculated.

Chemical examinations included colour reactions and thin-layer chromatography (TLC). Spot test reactions of thalli, apothecial margins and discs were made with KOH, sodium hypochlorite [commercial laundry bleach] (C) and paraphenylenediamine [solution in 95% ethyl alcohol] (PD). TLC analyses were performed in solvent systems A, B and C using the standardized method of Culberson (1972) and following Orange, James & White (2001).

Descriptions of the species are based on our own observations, measurements and TLC analyses of specimens cited in this paper. The terminology used in the descriptions of the species follows Ryan et al. (2004).

### Molecular methods: DNA extraction, PCR amplification and DNA sequencing

To infer relationships between species of lichenized fungi studied, the ITS rDNA region, that contains ITS1, 5.8S, ITS2 sequences, was used. Total DNA was extracted from specimens using a CTAB method (Doyle & Doyle, 1987). Dried samples were mechanically disintegrated using Mixer Mill MM400 (Retsch; Haan, Germany). The quality of the DNA was checked by electrophoresis on agarose gel (1%) with Simply Safe staining chemical (Eurx, Gdańsk, Poland). The complete ITS rDNA region was amplified using primers ITS1F (Gardes & Bruns, 1993) and ITS4 (White et al., 1990). The PCR reaction mix included 1U Taq recombinant polymerase (Thermo-Fisher Scientific, Waltham, USA), 10x Taq Buffer, 1 mM MgCl_2_, 0.5 M of each primer, 0.4 mM dNTP and 1 µl DNA template. Amplification cycles were performed with a Veriti Thermal Cycler (Life Technologies, Carlsbad, USA) and involved 8 min at 95 °C, followed by 32 cycles for 45 s at 95 °C and 45 s at 52 °C (annealing), and 1 min at 72 °C, with the a final extension step of 10 min at 72 °C. Amplified PCR products were purified using GeneJet PCR Purification Kit (Thermo-Fisher Scientific, Waltham, USA). This was accomplished at the Laboratory of Molecular Biology (Environmental and Life Sciences) at Wroclaw University. Sequencing of PCR products, post-reaction purification, forward and reverse directions reading were done by sequencing service Genomed (Genomed, Warsaw, Poland), using an ABI 377XL Automated DNA Sequencer (Applied Biosystems, Carlsbad, USA).

### Alignment assembly and molecular phylogenetic analyses

The obtained ITS rDNA sequences were assembled and manually edited using Geneious Pro, version 8.0. (Biomatters Ltd). Sequences of *Rhizoplaca subdiscrepans* (putative name for our collection) together with related species, namely *Lecanora pseudomellea, Protoparmeliopsis garovaglii, P. muralis, Rhizoplaca chrysoleuca, R. melanophthalma, R. novomexicana, R. occulta, R. opiniconensis* (as *Lecanora* in GeneBank), *R. parilis* and *R. phaedrophthalma*,** were included in the analysis. Our final ITS data-set included 21 sequences newly generated for this study and 95 sequences downloaded from GenBank (Table 1). The final alignment was performed on Geneious Pro using the MAFFT algorithm (Katoh et al., 2005), then re-checked and improved. Ambiguously aligned regions were removed in GBlocks (Castresana, 2000). The nucleotide substitution models were separately searched for in each partition of the ITS region (ITS1, 5.8S, ITS2) to find the best-fitting model using the corrected Akaike information criterion (AICc) as an optimality model criterion in a greedy algorithm search as implemented in PartitionFinder version 1.0.1 (Lanfear et al., 2012). The GTR+G model for ITS1 and ITS2 and K80 for the 5.8S partitions were selected.

The phylogenetic reconstruction was generated using the CIPRES Scientific Gateway (http://www.phylo.org/portal2/) (Miller, Pfeiffer & Schwartz, 2010). Maximum likelihood (ML) bootstrap tree with simultaneous heuristic search was undertaken as implemented in RAxML–HPC2 on XSEDE (Stamatakis, 2006) under the GTRGAMMA substitution model and 1,000 bootstrap resamples. Bayesian inference was carried out using Markov Chain Monte Carlo (MCMC) implemented in MrBayes 3.2.6 on XSEDE (Ronquist et al., 2012). MrBayes was set to two independent parallel runs each initiated with four incrementally heated chains; the run length was settled to 20M generations, and to infer convergence the average standard deviation of split frequencies was printed every 1000th generation discarding the first 50% of the trees sampled as a burn-in fraction. The analyses were stopped after 1M generations when the standard deviation had dropped below 0.01. The resulting phylogenetic trees were visualized in Figtree software (Rambaut, 2014). The alignment of ITS sequences described here is deposited in TreeBASE with number TB2: S26365.

## Results

Results of the morphological and chemical studies are presented in the taxonomic section under particular species descriptions. All taxa representing *R. subdiscrepans* s. lat. appear to be semi-cryptic, with slight morphological variety and some overlapping features. Nevertheless, some noticeable differences are visible in their chemistry, appearance of apothecia and marginal lobes, as well as in the shape and size of ascospores and conidia. The major distinguishing characters of *Rhizoplaca subdiscrepans, R. opiniconensis, R. phaedrophthalma,* and *R. pseudomellea* are summarized in Table 2.

**Table 1:** The species and specimens treated in current study with locality and GenBank accession numbers. Newly generated sequences are in boldface.

**Species**	**Locality**	**GenBank (ITS)** Accesion number
*Protoparmeliopsis garovaglii* 1	Austria	AF189718
*Protoparmeliopsis garovaglii* 2	USA, Utah, *Leavitt 199* (BRY-C)	KU934537
*Protoparmeliopsis garovaglii* 3	USA, Idaho, *Leavitt 078* (BRY-C)	KU934540
*Protoparmeliopsis garovaglii* 4	Poland, *Szczepańska 1240* (KRAM), L21	MK084624
*Protoparmeliopsis garovaglii* 5	Bolivia, *Flakus 17529* (KRAM), L88	MK084625
*Protoparmeliopsis garovaglii* 6	Bolivia, *Flakus 21175* (KRAM), L89	MK084626
*Protoparmeliopsis garovaglii* 7	Bolivia, *Flakus 21118* (KRAM), L90	MK084627
*Protoparmeliopsis garovaglii* 8	Peru, *Flakus 9603* (KRAM), L92	MK084628
*Protoparmeliopsis garovaglii* 9	Peru, *Flakus 9540* (KRAM), L91	MK084629
*Protoparmeliopsis garovaglii* 10	USA, Idaho, *Leavitt 109* (BRY-C)	KU934549
*Protoparmeliopsis muralis* 1	Romania, *J.-S. Hur* (KOLRI), SK 765	KP059048
***Protoparmeliopsis muralis* 2**	Czech Republic, Lipno nad Vltavou, Szczepańska 1263, isolate L97	**MN931719**
*Protoparmeliopsis muralis* 3	USA, Utah, *Leavitt 143* (BRY-C)	KT453726
*Protoparmeliopsis_muralis* 4	Germany, Saxony, *Scholz barcode M0275697* (M), DNA 9890	KT818623
*Protoparmeliopsis muralis* 5	USA, Utah, *Leavitt 077* (BRY-C)	KU934552
*Protoparmeliopsis muralis* 6	Russia, Chelyabinsk, *Vondrák 9405* (PRA)	KU934556
*Protoparmeliopsis muralis* 7	Russia, Kizilskoe, *Vondrák 9417* (PRA)	KU934557
*Protoparmeliopsis muralis* 8	Russia, Chelyabinsk, *Vondrák 9414* (PRA)	KU934558
*Protoparmeliopsis muralis* 9	Russia, Orenburg, *Vondrák 106b* (PRA)	KU934560
*Rhizoplaca melanophthalma* 1	USA, Nevada, LLS (EA 15-123A), BRY C55051	HM577272
*Rhizoplaca melanophthalma* 2	USA, Utah, LLS (EA 18-143), BRY C55052	HM577273
*Rhizoplaca melanophthalma* 3	USA, Colorado, LLS (EA 18-145), BRY C550	HM577274
*Rhizoplaca melanophthalma* 4	USA, Utah, LLS (EA 22-177), BRY C55054	HM577275
*Rhizoplaca melanophthalma* 5	USA, Idaho, LLS (EA 41-403), BRY C55055	HM577276
*Rhizoplaca melanophthalma* 6	USA, Wyoming, SDL, BRY C55056	HM577277
*Rhizoplaca melanophthalma* 7	USA, Wyoming, SDL, BRY C55057	HM577278
*Rhizoplaca melanophthalma* 8	USA, Utah, SDL, LLS, GS, BRY C55058	HM577279
*Rhizoplaca novomexicana* 1	USA, Utah, SDL, LLS, GS, BRY C55024	HM577255
*Rhizoplaca novomexicana* 2	USA, Utah, SDL, LLS, GS, BRY C55025	HM577256
*Rhizoplaca novomexicana* 3	USA, Utah, SDL, LLS, GS, BRY C55026	HM577257
*Rhizoplaca occulta* 1	USA, Utah, Juab Co.: West of Goshen, LLS (EA 18-140), BRY C55074	HM577305
*Rhizoplaca occulta* 2	USA, Idaho, Butte Co.: placeSalmon Challis National Forest, LLS (EA 37-356), BRY C55075	HM577306
*Rhizoplaca occulta* 3	USA, Nevada, White Pine Co.: Humboldt-Toiyabe N.F., SDL, LLS, BRY C55076	HM577307
*Rhizoplaca occulta* 4	USA, Idah, NA	AF159942
*Rhizoplaca occulta* 5	USA, Idaho, NA	AF159944
*Rhizoplaca chrysoleuca A*1	Russia, *Vondrák 9979* (PRA)	KU934563
*Rhizoplaca chrysoleuca A*2	Russia, *Vondrák 10021* (PRA)	KU934564
*Rhizoplaca chrysoleuca A*3	Russia, *Vondrák 10126* (PRA)	KU934566
*Rhizoplaca chrysoleuca A*4	Russia, *Vondrák 10040* (PRA)	KU934567
*Rhizoplaca chrysoleuca B*1	Russia, Altay, *Vondrák 9981* (PRA)	KU934568
*Rhizoplaca chrysoleuca B*2	Russia, Altay, *Vondrák 9993* (PRA)	KU934569
*Rhizoplaca chrysoleuca B*3	Russia, Altay, *Vondrák 10023* (PRA)	KU934570
*Rhizoplaca chrysoleuca B*4	Russia, Altay, *Vondrák 10059* (PRA)	KU934572
*Rhizoplaca chrysoleuca C*	Russia, Altay, *Vondrák 10017* (PRA)	KU934573
*Rhizoplaca opiniconensis* 1	USA, Arizona, Apache Co., *Nash III 27196 & Ryan* (ASU)	AF159928
*Rhizoplaca opiniconensis* 2	USA, Wisconsin, *Leavitt 12-005* (F)	KU934881
*Rhizoplaca opiniconensis* 3	USA, Wisconsin, *Leavitt 12-007* (F)	KU934882
*Rhizoplaca opiniconensis* 4	Russia, Altay, *Vondrák 10013* (PRA)	KU934883
*Rhizoplaca opiniconensis* 5	Russia, Altay, *Vondrák 10029* (PRA)	KU934884
*Rhizoplaca opiniconensis* 6	Russia, Altay, *Vondrák 10034* (PRA)	KU934885
*Rhizoplaca opiniconensis* 7	Russia, Altay, *Vondrák 10128* (PRA)	KU934886
***Rhizoplaca opiniconensis* 8**	Canada, Ontario, Leeds Co., *Brodo 25117* (CANL, holotype)	**MN931720**
***Rhizoplaca opiniconensis* 9**	Canada, Ontario, *Wetmore 88134* (MIN), isolate L30	**MN931721**
***Rhizoplaca opiniconensis* 10**	USA, Minnesota, Otter Tail Co., *Wetmore 74475* (MIN), isolate L35	**MN931722**
*Rhizoplaca parilis* 1	USA, Utah, LDP, BRY C55077	HM577308
*Rhizoplaca parilis 2*	USA, Utah, LDP, BRY C55078	HM577309
*Rhizoplaca parilis* 3	USA, Utah, LDP, BRY C55079	HM577310
*Rhizoplaca parilis* 4	USA, Utah, LDP, BRY C55080	HM577311
*Rhizoplaca parilis* 5	USA, Utah, LDP, BRY C55081	HM577312
*Rhizoplaca phaedrophthalma* 1	China,Tibet, *s. coll, s. herb.*	AF159938
*Rhizoplaca phaedrophthalma* 2	USA, Utah, *Leavitt 734* (BRY-C)	HM577230
*Rhizoplaca phaedrophthalma* 3	USA, Utah, *Leavitt 735* (BRY-C)	HM577231
*Rhizoplaca phaedrophthalma* 4	USA, Utah, *Leavitt 1023* (BRY-C)	HM577232
*Rhizoplaca phaedrophthalma* 5	Russia, state unknown, *Vondrák 9406 & Frolov* (PRA)^a^	KU934870
*Rhizoplaca phaedrophthalma* 6	Russia, state unknown, *Vondrák 9406* (PRA)	KU934871
*Rhizoplaca phaedrophthalma* 7	USA, Nevada, *Leavitt 9052* (F)	KU934872
*Rhizoplaca phaedrophthalma* 8	Russia, Orenburg, *Vondrák 9384* (PRA)	KU934873
*Rhizoplaca phaedrophthalma* 9	Russia, Orenburg, *Vondrák 9406* (PRA)	KU934875
*Rhizoplaca phaedrophthalma* 10	Russia, Chelyabinsk, *Vondrák 9405* (PRA)	KU934876
***Rhizoplaca phaedrophthalma* 11**	Canada, British Columbia, *Ryan 31877* (ASU), isolate L48	**MN931728**
***Rhizoplaca phaedrophthalma* 12**	Canada, British Columbia, *Ryan 31889* (ASU), isolate L49	**MN931729**
***Rhizoplaca phaedrophthalma* 13**	USA, Idaho, Twin Falls Co., *Ryan 32870* (ASU), isolate L50	**MN931730**
***Rhizoplaca phaedrophthalma* 14**	USA, Montana, Gallatin Co., *Wetmore 80571* (MIN), isolate Lec10	**MN931732**
***Rhizoplaca phaedrophthalma* 15**	USA, Montana, Park Co., *Wetmore 80979* (MIN), isolate L16	**MN931726**
***Rhizoplaca phaedrophthalma* 16**	USA, Oregon, Deschutes Co., *Wetmore 95059* (MIN), isolate L12	**MN931723**
***Rhizoplaca phaedrophthalma* 17**	USA, Oregon, Malheur Co., *Wetmore 5101* (MIN), isolate L14	**MN931724**
***Rhizoplaca phaedrophthalma* 18**	USA, Wyoming, Park Co., *Wetmore 80866* (MIN), isolate Lec9	**MN931731**
***Rhizoplaca phaedrophthalma* 19**	USA, Wyoming, Park Co., *Wetmore 81235* (MIN), isolate L15	**MN931725**
***Rhizoplaca phaedrophthalma* 20**	USA, Wyoming, Park Co., *Wetmore 81446* (MIN), isolate L17	**MN931727**
*Rhizoplaca polymorpha* 1	USA, Utah, LDP, BRY C55091	HM577322
*Rhizoplaca polymorpha* 2	USA, Utah, LDP, BRY C55092	HM577323
*Rhizoplaca polymorpha* 3	USA, Idaho, SDL, HCL, JHL, BRY C55094	HM577325
*Rhizoplaca polymorpha* 4	USA, Idaho, SDL, HCL, JHL, BRY C55095	HM577326
*Rhizoplaca polymorpha* 5	USA, Utah, SDL et al., F 11-026	JX948194
***Rhizoplaca pseudomellea* 1**	USA, California,Tulare Co., *Wetmore 51151* (MIN), isolate L93	**MN931734**
***Rhizoplaca pseudomellea* 2**	USA, California, Tuolumne Co., *Ryan 24402* (MIN), isolate L94	**MN931735**
***Rhizoplaca pseudomellea* 3**	USA, Oregon, Harney Co., *Wetmore 95079* (MIN), isolate Lec7	**MN931736**
***Rhizoplaca pseudomellea* 4**	USA, Oregon, Harney Co., *Wetmore 95084* (MIN), isolate Lec8	**MN931737**
***Rhizoplaca pseudomellea* 5**	USA, Oregon, Lake Co., *Ryan 28456* (ASU), isolate L40	**MN931733**
*Rhizoplaca subdiscrepas B*1	China, Yanbian Korean Autonomous Prefecture, *s. coll, s. herb.*	KP226212
*Rhizoplaca subdiscrepans B*2	Kazahztan, Karkaralinsk, *Seaward s.n.* (BRY-C)	KU934877
*Rhizoplaca subdiscrepans B*3	Russia, Altay, *Vondrák 10001* (PRA)	KU934878
*Rhizoplaca subdiscrepans B*4	Russia, Chelyabinsk, *Vondrák 10049* (PRA)	KU934879
*Rhizoplaca subdiscrepnas C*	Russia, Altay, *Vondrák 9975* (PRA)	KU934880
*Rhizoplaca subdiscrepans s.s.*1	Russia, state unknown, *Vondrák jv5* (PRA)	KU934887
*Rhizoplaca subdiscrepans s.s.*2	Russia, Orenburg, *Vondrák 440_Irikla4* (PRA)	KU934888
*Rhizoplaca subdiscrepans s.s.*3	Russia, Orenburg, *Vondrák 442_Irikla4* (PRA)	KU934889
*Rhizoplaca subdiscrepans s.s.*4	Russia, Orenburg, *Vondrák 9384 & Frolov* (PRA)^a^	KU934890
*Rhizoplaca subdiscrepans s.s.*5	Russia, Orenburg, *Vondrák 9385 & Frolov* (PRA)^a^	KU934891
*Rhizoplaca subdiscrepans s.s.*6	Russia, Bashkortostan, *Vondrák 9411 & Frolov* (PRA)^a^	KU934892
*Rhizoplaca subdiscrepans s.s.*7	Russia, Orenburg, *Vondrák 9415 & Frolov* (PRA)^a^	KU934893
*Rhizoplaca subdiscrepans s.s.*8	Russia, Orenburg, *Vondrák 9416 & Frolov* (PRA)^a^	KU934894
*Rhizoplaca subdiscrepans s.s.*9	Russia, Chelyabinsk, *Vondrák 9418 & Vondráková* (PRA)^a^	KU934895
*Rhizoplaca subdiscrepans s.s.*10	Russia, Orenburg, *Vondrák 9422a & Frolov* (PRA)^a^	KU934896
*Rhizoplaca subdiscrepans s.s.*11	Russia, Orenburg, *Vondrák 9422b & Frolov* (PRA)^a^	KU934897
*Rhizoplaca subdiscrepans s.s.*12	Russia, Chelyabinsk, *Vondrák 9408 & Vondráková* (PRA)^a^	KU934898
*Rhizoplaca subdiscrepans s.s.*13	Russia, Orenburg, *Vondrák 9412 & Vondrákov*á (PRA)^a^	KU934899
*Rhizoplaca subdiscrepans s.s.*14	Russia, Orenburg, *Vondrák 9420 & Frolov* (PRA)^a^	KU934900
*Rhizoplaca subdiscrepans s.s.*15	Russia, Orenburg, *Vondrák 9420b & Frolov* (PRA)^a^	KU934901
*Rhizoplaca subdiscrepans s.s.*16	Russia, Orenburg, *Vondrák 9420c & Frolov* (PRA)^a^	KU934902
*Rhizoplaca subdiscrepans s.s.*17	Russia, Orenburg, *Vondrák 9420d & Frolov* (PRA)^a^	KU934903
*Rhizoplaca subdiscrepans s.s.*18	Ukraine, Prague, *Vondrák 9843* (PRA)	KU934904
*Rhizoplaca subdiscrepans s.s.*19	Russia, state unknown, *Vondrák jv4* (PRA)	KU934905
*Rhizoplaca subdiscrepans s.s.*20	Russia, Orengurg, *Vondrák 9384a* (PRA)	KU934906
***Rhizoplaca subdiscrepans s.s.*21**	Poland, Sudety Mts foreland, *Szczepańska 923* (KRAM), isolate 11pLecanora_new	**MN931738**
***Rhizoplaca subdiscrepans s.s.*22**	Poland, Sudety Mts foreland, *Szczepańska 967* (KRAM), isolate 13pLecanora_new	**MN931739**

**Notes.**

a Specimens examined (available for study in PRA).

**Figure 1:**
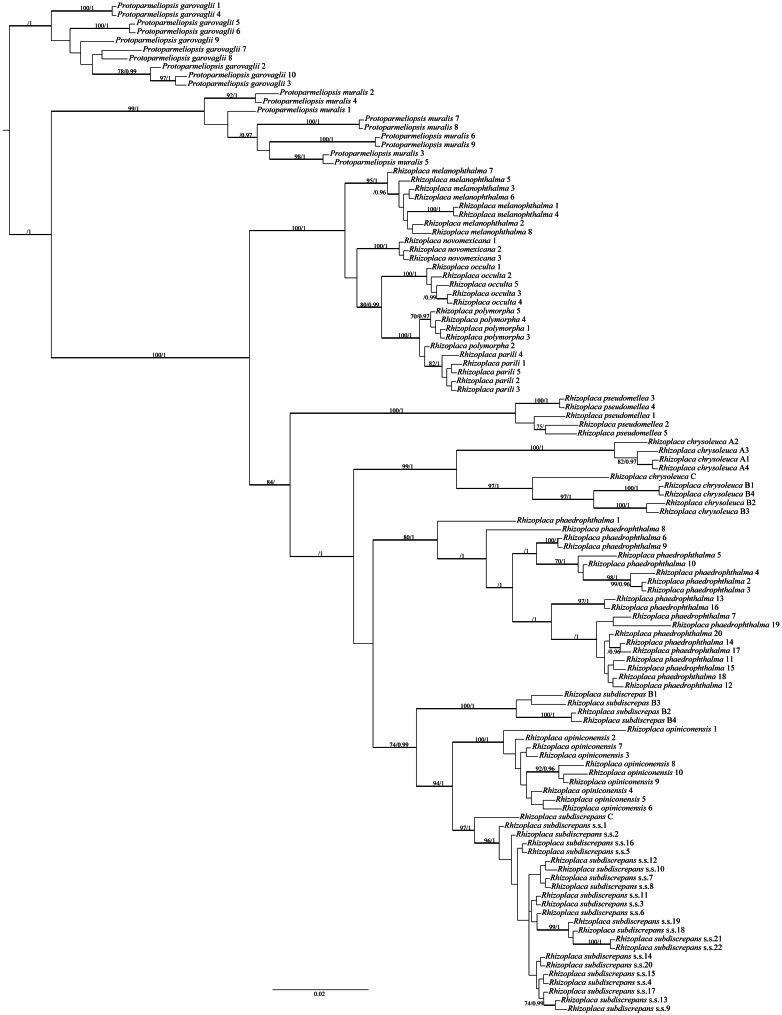
Bayesian inference of phylogenetic relationship within *Rhizoplaca subdiscrepans* s. lat.** based on ITS rDNA sequences. High bootstrap support values are shown above thickened branches and bold numbers representing clades (ML –BP ≥ 70%, Bayesian analysis –PP ≥ 0.9).

**Figure 2:**
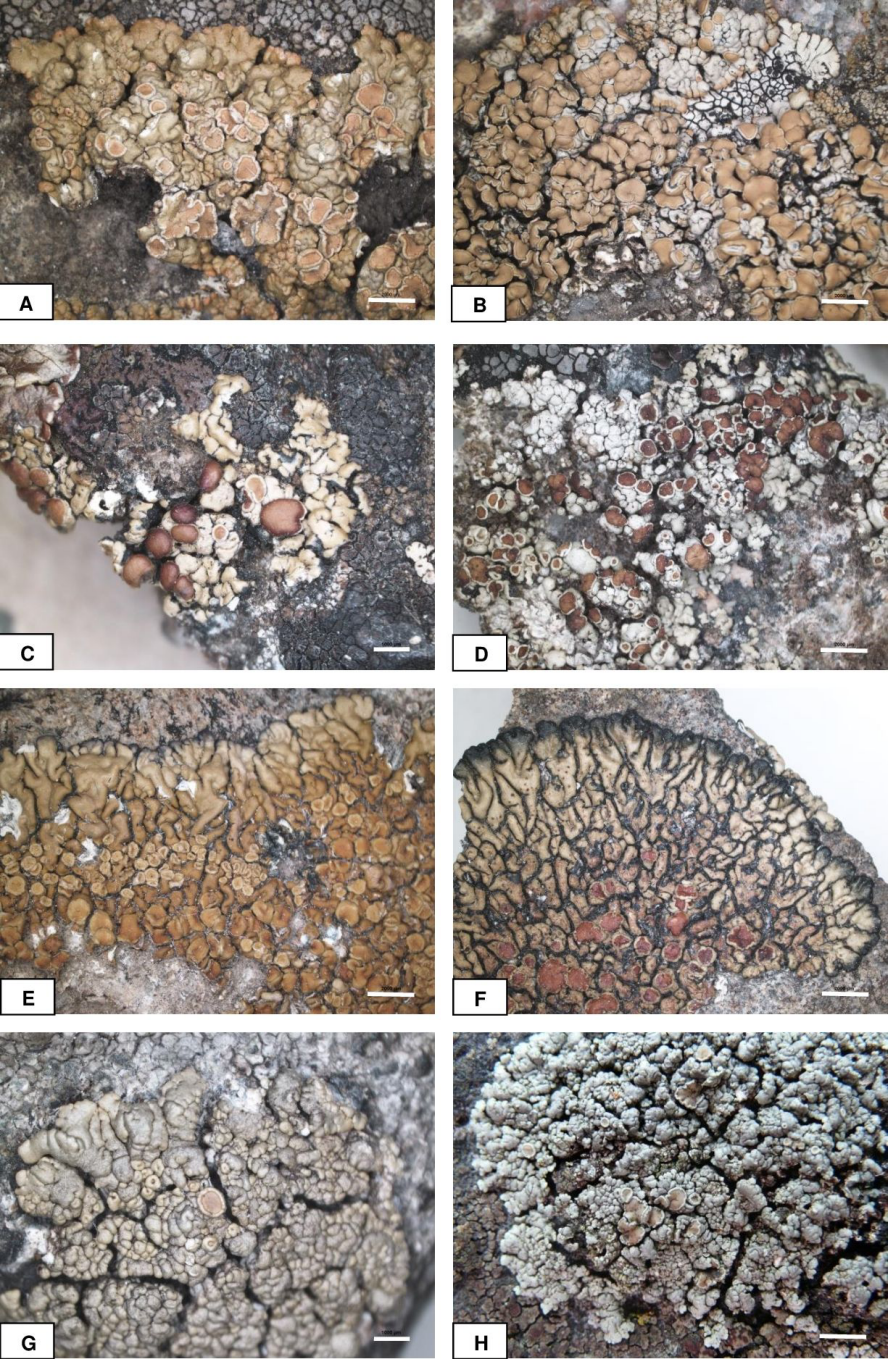
Habitus of *Rhizoplaca* species treated. Habitus of *Rhizoplaca* species treated. (A) *R. opiniconensis, I.M. Brodo 25117*, CANL (holotype). (B) *R. opiniconensis*, *J. Schuster 3098*, ASU. (C) *R. phaedrophthalma, F. Lobbichler, M* 122-86/40 (holotype). (D) *R. phaedrophthalma*, *C.M. Wetmore 81235*, MIN. (E) *R. pseudomellea, B.D. Ryan 13609*, ASU (holotype). (F) *R. pseudomellea*, *C.M. Wetmore 95084*, MIN. (G) *R. subdiscrepans, K. Szczepańska 967*, WRSL. (H) Thallus of *R. subdiscrepans* in natural habitat. Bars = one mm (C, G), two mm (A–B, D–F, H). (Photos: K. Szczepańska, K. Wilk).

The final alignment matrix contained 538 bp and *Protoparmeliopsis* was selected as the outgroup (Leavitt et al., 2016; Zhao et al., 2016). The topology of the tree was similar to that presented by Leavitt et al. (2016). Our newly generated sequences from Poland, Canada and the USA were resolved within *R. subdiscrepans* s. lat. as defined by Leavitt et al. (2016) (Fig. 1). The first group of samples (*Rhizoplaca subdiscrepans* s. str. 21 and 22) were recovered within the clade ‘subd E’ with high bootstrap support (BS) = 96 and posterior probability (PP) = 1 together with specimens from Ukraine and Russia (*Rhizoplaca subdiscrepans* s. str. 18 and 19 respectively). Sequences of herbarium specimens marked as *R. phaedrophthalma* were nested within the clade ‘subd A’ (BS = 80 and PP = 1), together with the sequence of *R. phaedrophthalma* published by Arup & Grube (2000) and Zhao et al. (2016), while *R. opiniconensis* (BS = 100 and PP = 1) nested within the clade ‘subd D’ of Leavitt et al. (2016). Moreover, the sequence of the type collection of *R. opiniconensis* (*Rhizoplaca opiniconensis* 10; see Fig. 2A), another sample identified by Irwin Brodo, who described *R. opiniconensis* as a new taxon (Brodo, 1986), as well as the sequence of *R. opiniconensis* analysed by Arup & Grube (2000) and Zhao et al. (2016)*,* were placed within the lineage of candidate species ‘subd D’. *Lecanora pseudomellea*, included for the first time in a phylogenetic analysis of *Rhizoplaca*, formed a strongly supported monophyletic lineage (BS = 100 and PP = 1) within the latter genus.

**Table 2:** Overview of the distinguishing features of the *Rhizoplaca subdiscrepans* s. str., *R. opiniconensis, R. phaedrophthalma and R. pseudomellea*.

Character	Species			
	*R. subdiscrepans s.s.* (*R. subdiscrepans* E’)^a^	*R. opiniconensis* (*R. subdiscrepans* D’)^a^	*R. phaedrophthalma* (*R. subdiscrepans* A’)^a^	*R. pseudomellea*
Thallus morphology	polyphyllous placodioid, yellow-green, glossy marginal lobes distinctly developed, discrete or contiguous to slightly overlapping, convex, broadened, flabellate and crenate-incised at tips thallus centre squamulose-bullate, composed of convex subunits squamules convex, sinusoid and plicate	polyphyllous, placodioid, yellow-green, with orange tint in older collections, dull	polyphyllous placodioid, yellow-green, dull	placodioid, orange-brown to reddish or rusty brown, glossy
		marginal lobes distinctly developed, slightly convex, broadened, flabellate and crenate-incised at tips	marginal lobes weakly developed, slightly convex, broadened and flabellate at tips	marginal lobes distinctly developed, long, slightly convex, minutely broadened and greenish-black to black at tips
		thallus centre squamulose-areolate to squamulose-bullate	thallus centre squamulose-areolate to squamulose-bullate	thallus center areolate
		squamules flat to strongly convex, irregular	squamules flat to slightly convex, irregular	areoles convex, rounded to irregular
Apothecia	sessile, disc pale yellowish-brown, plane, epruinose, margin persistent	sessile to constricted, disc pale yellowish-brown, plane, epruinose, margin persistent	sessile, disc reddish-brown, strongly convex, epruinose, margin excluded in older apothecia	sessile, disc orange-brown to reddish-brown, plane, epruinose margin excluded in older apothecia
Ascospores	ellipsoid 10–12 × 6–7 μ m	ellipsoid 10–12 × 6–7 μ m	subglobose 8–10 × 5–7 μ m	ellipsoid 10–12 × 6–7 μ m
Conidia	filiform, mostly straight 10–25 × 0.5 μ m	filiform, mostly arched 15–25 × 0.5 μ m	filiform, mostly straight 15–30 × 0.5 μ m	filiform, straight 10–25 × 0.5 μ m
Chemistry	isousnic (+∕ −), usnic (+), placodiolic (+), fatty acids (+∕ −), terpenoids (+)	isousnic (+∕ −), usnic (+), placodiolic (+), terpenoids (+∕ −)	usnic (+), placodiolic (+), terpenoids (+∕ −)	isousnic (+∕ −), usnic (+∕ −), psoromic (+∕ −), fatty acids (+∕ −)
Ecology	siliceous rock at lower elevations, in dry and sunny sites	siliceous rock at higher elevations, in shaded sites near streams	siliceous rock at higher elevations, in semi-arid areas	siliceous rock at higher elevations, in dry and sunny sites
Geography	Europe and Western Asia	North America and East-Central Asia	North America and Westen and Central Asia	North America
*Locus classicus*	Europe (Switzerland)	North America (Canada)	Asia (Nepal)	North America (USA)

**Notes.**

a Candidate species by Leavitt et al. (2016).

### Taxonomy

***Rhizoplaca opiniconensis*** (Brodo) Leavitt, Zhao Xin & Lumbsch, in Zhao et al., Fungal Diversity 78: 302. 2016. ≡ *Lecanora opiniconensis* Brodo, Mycotaxon 26: 309. 1986.

Figs. 2A, 2B.

**Type**: [CANADA], ONTARIO: Leeds Co., Snake Island, in Lake Opinicon, Queen’s University Biological Station, Chaffeys Locks, on S-facing rockface, 1.0–1.5 m above water, 2 Feb. 1985, *I.M. Brodo 25117* (holotype, CANL!).

*Thallus* lichenized, placodioid, polyphyllous, usually distinctly rounded, irregular when older, thick, loosely attached to the substrate, 2–5 cm diam. or more. Marginal lobes distinctly developed, short, 0.5–1 mm long and 0.2–0.6 mm wide, plane to slightly convex, broadened and crenate-incised at the tips. Thallus centre squamulose-areolate to squamulose-bullate, irregularly cracked, thick, loosely attached to the substrate. Squamules crowded, flat to strongly convex, irregular, 0.2–1.5 mm diam. Upper surface smooth, dull, yellowish-green to yellowish-orange (more evident in herbarium material), marginal lobes usually darker than the thallus centre. *Apothecia* numerous, sessile to constricted, 0.2–2 mm diam. circular, older apothecia irregular and distorted. Margin thin, concolourous with thallus or paler, flexuose, crenate, usually persistent, sometimes excluded, disc pale yellowish-brown to yellowish-orange, epruinose, plane, rarely convex, dull. *Hymenium* colourless, 60–70 μm high, hypothecium colourless. *Epihymenium* orange-brown to olive-brown, with small granules dissolving in K. *Ascospores* 8 per ascus, hyaline, simple, ellipsoid, 10–12 × 6–7 μm. *Pycnidia* immersed, simple with bluish-black ostioles, conidia hyaline, filiform, mostly arched 15–25 × 0.5 μm.

**Chemistry***.* Thallus K–, C–, KC+ yellow, PD–, medulla K+ yellow, C–, KC+ yellow, PD–. Secondary metabolites detected by TLC: isousnic (+∕ −), usnic (+) and placodiolic (+) acids, as well as some unidentified terpenoids (+∕ −).

**Ecology and distribution***.* On quartzite and volcanic rocks, at higher elevations (c. 1,200–3,000 m), in shaded places near streams, in North America and Eastern Asia.

**Comments**. Characteristic features of *R. opiniconensis* are the mostly arched conidia and thallus colour, which changes to a more orange colour in herbarium specimens. Some specimens are morphologically similar to *R. subdiscrepans* s. str., in which case proper identification of both taxa may be difficult. However, in contrast to *R. subdiscrepans* s. str., *R. opiniconensis* does not produce fatty acids and was genetically confirmed in North American and Asian but not from European specimens. Additionally, the species has also specific habitat preferences.

**Specimens examined.** CANADA. ONTARIO: West edge of Marathon, shore of Lake Superior and back on rock ridges with black spruce and scattered quaking aspen, alt. 629 ft, 5 Aug. 2002, *C.M. Wetmore 88134* (MIN). MEXICO. MEXICO: Baja California Sur, below top of Sierra Agua Verde (part of the Sierra San Francisco), on rhyolite, N-facing cliff, alt. c. 1300 m, 1 Jan. 1998, *T.H. Nash III 40100* (ASU); Chihuahua, along secondary dirt road to Casa Grandes from Bavispe, Sonora, oak woodland area with large outcrops of rhyolite, on rhyolite, alt. c. 2050 m, 18 July 1994, *T.H Nash III 36458* (ASU). USA. ARIZONA: Apache Co., Apache National Forest, Mt Baldy Wilderness area, trail from Phelp’s cabin along East Fork of Little Colorado River, on rock, alt. 3001 m, 1 July 1990, *T.H Nash III 27051* (ASU); Apache Co., Petrified Forest National Park, south of I-40, east of Petrified Forest Rd, near Crystal Forest Trail, on sandstone, alt. 1669 m, 14 May 1990, *W.C. Davis 735* (ASU); Cochise Co., Chiricahua Mountains, Colorado National Forest, cliff area above Mormon Springs in Mormon Canyon, oak woodland, on exposed cliff-face, alt. c. 2070 m, 24 Nov. 1995, *T.H. Nash III 37156* (ASU); Cochise Co., Chiricahua Mountains, Chiricahua National Monument, along the Loop Trail above Rhyolite Canyon and below “Heart of the Rocks”, oak-pine forest, on rhyolite, alt. c. 2000 m, 5 June 1998, *T.H. Nash III 41767* (ASU); Cochise Co., Chiricahua Mountains, Chiricahua National Monument, just before junction of trail to Heart of Rocks and trail to Massai Point, steep NE-facing part of rhyolite boulders under tall rock formation, alt. c. 2020 m, 5 June 1993, *B.D. Ryan 30861* (ASU); Graham Co., E side of the Pinaleno Mountains, along trail above Shannon Campground, mixed conifer-oak forest, on acidic rock, alt. c. 2000 m, 15 July 1994, *T.H Nash III 36056* (ASU); Pima Co., east side of Baboquivari Peak, on exposed granite, alt. c. 1920 m, 13 Oct. 1990, *T.H. Nash III 27444* (ASU); Pima Co., Santa Catalina Mountains, Coronado National Forest, upper part of Sabino Canyon, near Marshall Gulch, granite boulders in mixed conifer forest, on granite, alt. c. 2310 m, 3 June 1998, *T.H. Nash III 41709* (ASU). MINNESOTA: Cook Co., Grand Portage State Forest, Lake Superior shore NE of Horseshoe Bay (16 miles SW of Grand Portage), shrubby wet cobble shore, alt. 603 ft, 28 Sept. 2001, *C. Reschke 1567* (MIN); Cook Co., Grand Portage National Monument, north side of Mt Rose, rock cliffs and large rock blocks below with some white birch and white spruce, alt. 867 ft, 13 July 2012, *C.M. Wetmore 100545* (MIN); Pope Co., Glacial Lakes State Park, 5 miles S of Starbuck, Prairie area with border woods on esker 0.1 mile NE of Mountain Lake, 7 Aug. 1993, *J.P. Schuster 3098* (ASU); Lake of the Woods Co., Clementson (8 miles east of Baudette), at mouth of Rapid River to Rainy River, on hillside with bur oak, ash, white birch and balsam poplar, also on rocks above rapids, 18 Aug. 1994, *C.M. Wetmore 74873* (MIN); Otter Tail Co., Inspiration Peak State Scenic Wayside Park, 12 miles west of Parkers Prairie, on hill with grass and rock on top and on hillside with red oak and bur oak, 14 Aug. 1994, *C.M. Wetmore 74475* (MIN); Washington Co., Lost Valley Prairie Scientific and Natural Area, prairie area with limestone outcroppings and cedar trees, surrounded by aspen and mixed hardwoods, 3 miles NNW of junction of St Croix and Mississippi Rivers, 3 miles NNE of Hastings, 5 Aug. 1990, *J.P. Schuster 2460* (KRAM). NEW MEXICO: Cibola Co, Cibola National Forest, Zuni Canyon 0.2 miles SW of Log Chute (8.5 miles SW of Grants), small N-S canyon with rocks, douglas fir, oak and few ponderosa pine, alt. 7434 ft, 17 June 2010, *C.M. Wetmore 99445* (MIN); Cibola Co., El Malpais National Conservation Area above Ranger Station (12.8 miles S of Grants), ridge S of ranger station with juniper, sage and rocks, alt. 6837 ft, 15 June 2010, *C.M. Wetmore 99205* (KRAM); Cibola Co, El Malpais National Conservation Area, 9 miles S of Grants, lava beds with scattered juniper, alt. 6553 ft, 15 June 2010, *C.M. Wetmore 99234* (MIN); McKinley Co., Cibola National Forest, 0.5 mile E of Quaking Aspen campground on USFS 162 (15 miles SE of Gallup), valley with rock ledges, ponderosa pine, juniper and oak, alt. 7735 ft, 18 June 2010, *C.M. Wetmore 99527* (KRAM); San Miguel Co., near town of Villanueva (42 miles SW of Santa Fe), hillside and ridge near dam with juniper and pinyon pine, alt. 5900 ft, 11 June 2010, *C.M. Wetmore 98925* (KRAM).

***Rhizoplaca phaedrophthalma*** (Poelt) Leavitt, Zhao Xin & Lumbsch, in Zhao et al., Fungal Diversity 78: 302. 2016 ≡ *Lecanora phaedrophthalma* Poelt, Mitt. Bot. Staatss. München 2: 483. 1958.

Figs. 2C, 2D.

**Type**: [NEPAL], NÖRDL. ZENTRALNEPAL: Manangbhot (oberes Marsyandi –Tal) Felsen nördlich Banphag, alt. 4100 m, 13 June 1955, *Fr. Lobbichler* (holotype, M 122–86/40!).

*Thallus* lichenized, placodioid, polyphyllous, rounded to irregular when older, 2–5 cm diam. Marginal lobes usually weakly developed, 0.5–1.2 mm long and 0.2–0.7 mm wide, short, plane to slightly convex, broadened at the tips and flabellate. Thallus centre squamulose-areolate to squamulose-bullate, irregularly cracked, thick, loosely attached to the substrate. Squamules crowded, flat to slightly convex, irregular, 0.5–2 mm diam., usually with bluish-black tinges on the margins and underside towards edges. Upper surface smooth, dull, pale and light yellow, pruinose in parts, marginal lobes concolourous with thallus centre. *Apothecia* numerous, sessile, dispersed to grouped mainly in the centre, 0.5–1.2 mm diam., circular, older irregular. Margin thin, smooth, concolourous with thallus, slightly raised when young, then excluded, disc orange-brown to reddish-brown, usually contrasting with the thallus, epruinose, strongly convex, dull. *Hymenium* colourless 50–60 μm high, hypothecium colourless. *Epihymenium* orange-brown, with small granules dissolving in K. *Ascospores* 8 per ascus, hyaline, simple, subglobose, 8–10 × 5–7 μm. *Pycnidia* immersed, simple with bluish-black ostioles, conidia hyaline, filiform, mostly straight, 15–30 × 0.5 μm.

**Comments**. The main characteristic feature of *R. phaedrophthalma* is the morphology of apothecia, which are strongly convex, with reddish-brown, epruinose discs and excluded margin. Additionally, mature specimens of the species, in contrast to *R. opiniconensis* and *R. subdiscrepans* s. str., are usually poorly placodioid, with weakly define marginal lobes. *R. phaedrophthalma* seems to be also characterized by the size of its conidia, which are slightly larger than in other species treated, as well as by subglobose rather than ellipsoid spores. Concerning secondary metabolites produced by the species, neither isousnic acid nor fatty acids were seen in TLC, unlike from the thalli of other taxa.

**Specimens examined**. CANADA. BRITISH COLUMBIA: Buse Hill, on exposed rock near top of hill, alt. c. 950 m, 27 Aug. 1994, *B.D. Ryan 31877* (ASU); near Quilchena Hotel, alt. c. 625 m, 28 Aug. 1994, *B.D. Ryan 31889* (ASU). RUSSIA. ORENBURG REGION: Surroundings of water reservoir “Iriklinskoe vodokhranilishche”, Iriklinskiy, vill. Vishevoe, rocks 2 km E of village, in valley of stream, alt. 250–270 m, on acidic schist, 10 June 2011, *J. Vondrák 9406 & Frolov* (PRA, two samples). USA. COLORADO: Park Co., Pennsylvania Mt, Pike National Forest, on rock, c. 3688 m, 1 June 1990, *M.A. Thomas 32870* (ASU). IDAHO: Twin Falls Co., Hagerman Fossil Beds National Monument, 0.5–1 km E of S end of 500 East Rd, on basalt, alt. c. 900 m, 10 Sept 1998, *B.D. Ryan 32870* (ASU). MONTANA: Gallatin Co., Yellowstone National Park, below Black Butte along highway 191 near NW corner of park, on talus slope and along highway, alt. 6700 ft, 15 July 1998, *C.M. Wetmore 80571* (MIN); Park Co., Yellowstone National Park, Grazing enclosure 1 mile W of Garsiner at northern edge of park. Open grassland on knoll with sagebrush and rock outcrop, alt. 5300 ft, 21 July 1998, *C.M. Wetmore 80979* (MIN). OREGON: Deschutes Co., 6 miles W of Redmond, Juniper and sage area with lots of loose soil, alt. 3020 ft, 18 July 2006, *C.M. Wetmore 95059* (MIN); Malheur Co., along US20 above Malheur River 13 miles SW of Harper, Sage brush prairie on north slope, alt. 2790 ft, 19 July 2006, *C.M. Wetmore* 5101 (MIN). WYOMING: Park Co., West and of Lamar Canyon, 6 miles E of Tower Junction, on south facing hillside with large granitic glacial erratics and scattered douglas fir, alt. 6500 ft, 19 July 2006, *C.M. Wetmore 81235* (MIN); Park Co., Yellowstone National Park. Pebble Creek Trail at rocks above cliffs, below switchback, 0.25 miles up from camp-ground, alt. 6900 ft, 27 July 1998, *C.M*. *Wetmore 81446* (MIN); Park Co., Yellowstone National Park, Sheepeater Cliffs, 5.5 miles S of Mammoth, on south-facing columnar cliffs and talus above stream, alt. 7200 ft, 20 July 1998, *C.M. Wetmore 80866* (MIN).

***Rhizoplaca subdiscrepans*** (Nyl.) R. Sant., The Lichens of Sweden and Norway: 278. 1984. ≡ *Squamaria chrysoleuca* var. *subdiscrepans* Nyl., Flora 44: 718. 1861. ≡ *Lecanora subdiscrepans* (Nyl.) Stizenb., Berichtüber die Tätigkeit der St. Gallischen Naturwissenschaftlichen Gesellschaft 1880-1881: 341. 1882.

Figs. 2G, 2H.

**Type**: [EUROPE], Helvetiae et Tyroliae (PC).

*Thallus* lichenized, placodioid, polyphyllous, usually rounded, thick, convex, loosely attached to the substrate, 1–5 cm or more in diam. Marginal lobes distinctly developed, short, 1–2.5 mm long and 0.5–1.5 mm wide, discrete or contiguous to slightly overlapping, convex, broadened and crenate-incised at the tips. Thallus centre squamulose-bullate, composed of crowded strongly convex subunits. Squamules rounded, sinusoid and plicate, 0.25–1 mm diam. Upper surface smooth, glossy, greenish-yellow, in shade becoming greyish-green, marginal lobes concolourous with thallus centre, upper cortex interspersed with brownish granules soluble in K. *Apothecia* usually numerous and grouped, 0.5–2.5 mm diam., rounded, sessile, adnate. Margin prominent, rather thick, concolourous with thallus, shiny, entire to crenate when older, disc plane, bright orange, yellowish-orange to brownish-orange, matte, epruinose. *Hymenium* colourless, 50–80 μm high, hypothecium colourless, paraphyses simple or weakly branched with swollen apices, subhymenium well-differentiated, pale grey. *Epihymenium* orange-brown, interspersed with small granules dissolving in K. *Ascospores* 8 per ascus, hyaline, simple, ellipsoid, 11–12  ×  6–7 μm. *Pycnidia* immersed, simple with bluish-black ostioles, conidia hyaline, filiform, mostly straight 10–2 × 0.5 μm.

**Chemistry**. Thallus K+ yellow, C+ pale yellow, KC+ yellow, PD–, medulla K+ yellow, C+ yellow, KC+ yellow, PD–. Secondary metabolites detected by TLC: isousnic (+∕ −), usnic (+) and placodiolic (+) acids, as well as unidentified fatty acid (+∕ −) and unidentified terpenoids (+).

**Ecology and distribution**. On volcanic and granite rocks in warm and sunny places, at lower elevations (200–400 m), and very rare at higher elevations (c. 800 m), in Europe (Poland and Ukraine) and Western Asia.

**Comments**. Thallus morphology of specimens representing *R. subdiscrepans* s. str. is rather homogeneous and similar to *R. opiniconensis*. Both species have a placodioid thallus, squamulose-bullate in the centre, however *R. opiniconensis* usually has an orange tint to the thallus, especially visible in older collections. Nevertheless, *R. subdiscrepans* s. str. produces fatty acids that have not been found in other taxa of *R. subdiscrepans* s. lat.

**Specimens examined**. HUNGARY: Montes Matra, region montis Kékes, in rupibus Saskö dictis, on andesitic rock, alt. 880 m, 6 June 1974, *G. Kiszely & A. Vezda* (KRAM, two specimens). POLAND. SUDETY MTS: Przedgórze Sudeckie foreland, Wzgórza Strzegomskie Hills, Góra Krzyzowa Mt, alt. 354 m, on basalt rocks in open place, 4 Oct. 2013, *K. Szczepanska 923*, *967* (KRAM, hb. Szczepanska). RUSSIA. CHELYABINSK REGION: Kizilskoe, Obruchevka (c. 20 km E of Kizilskoe), Mt Razbornaya, c. 8 km W of village, alt. c. 450 m., on granite outcrops and stones, 25 June 2011, *J. Vondrák 9408 & Vondráková* (PRA); Kizilskoe, Obruchevka (c. 20 km E of Kizilskoe), Mt Razbornaya, c. 8 km W of village, alt. c. 450 m., on sun-exposed granite outcrops and stones, 25 June 2011, *J. Vondrák 9418 & Vondráková* (PRA). Orenburg region: surroundings of water reservoir “Iriklinskoe vodokhranilishche”, Iriklinskiy, vill. Chapaevka, volcanic and limestone rocks on opposite slope of lake W of village, alt. 270–290 m, on sunny volcanic rock, 11 June 2011, *J. Vondrák 9384, 9385, 9415 & Frolov* (PRA), 9422 *& Frolov* (PRA, two samples); Kuvandik, vill. Maloe Churaevo (c. 25 km N of Kuvandic) camp c. 2 km W of illage, steppes and *Quercus robur*-*Tilia cordata*-*Ulmus laevis* woodland areas around camp, alt. 250–500 m, on sun-exposed siliceous outcrops, 27 June 2011, *J. Vondrák 9412 & Vondráková* (PRA); surroundings of water reservoir “Iriklinskoe vodokhranilishche”, vill. Chapaevka, on opposite slope of lake, volcanic rock in valley of stream “Verkhnaya Orlovka”, alt. 270–300 m, on sun-exposed siliceous outcrops, 11 June 2011, *J. Vondrák 9416 & Frolov* (PRA), *9420 & Frolov* (PRA, four samples). REPUBLIC OF BASHKORTOSTAN: surroundings of water reservoir “Iriklinskoe vodokhranilishche”, vill. Tashtugay, rocks in valley of river Tanalik, c. 4 km S of village, alt. 260–300 m, on acid schist, 10 June 2011, *J. Vondrák 9411 & Frolov* (PRA). SWEDEN. Ngermanland: Vibyggeråpar., Värna, Valaberget (Mt c. 32 km SSE of Örnskoldsvik), steep southern slope facing the sea, on steep rocks to S, alt. 10 m, 11 Aug. 1986, *R. Moberg 6937* (KRAM).

### New combination

***Rhizoplaca pseudomellea*** (B.D. Ryan) Szczepanśka, Rodriguez-Flakus & Śliwa, comb. nov. ≡ *Lecanora pseudomellea* B.D. Ryan, Bryologist 96: 295. 1993.

MB 835038

Figs. 2E, 2F.

**Type**: [USA]. CALIFORNIA: Alpine Co., 1 mi E of Monitor Pass, along Calif. Hwy 89, 16 mi E of Markleeville, alt. 2440 m, field of small rocks among Sagebrush, 24 June 1985, *B.D. Ryan 13609* (holotype, ASU!).

*Thallus* lichenized, placodioid, usually distinctly rosette, moderately attached to the substrate, 1–4 cm diam. Marginal lobes distinctly developed, 1–3.5 mm long and 0.7–2 mm wide, slightly convex, smooth, contiguous, sinusoid, minutely broadened and grey, greenish-black to black at the tips. Thallus centre areolate, areoles convex, bullate, rounded to irregular, smooth or slightly rough, 0.5–2 mm diam. Upper surface smooth, glossy, yellowish-brown, orange-brown to reddish- or rusty brown, marginal lobes paler, yellowish, except darker tips. *Apothecia* grouped in the thallus centre, 0.5–2.5 mm diam., rounded to irregular when older, sessile. Margin thin, concolourous with thallus, shiny, flexuose, slightly raised when young, becoming excluded, disc concolourous with thallus or darker, reddish-brown, glossy, plane to slightly convex, epruinose. *Hymenium* colourless, 60–70 μm high, with colourless hypothecium. *Epihymenium* orange-brown, interspersed with small granules dissolving in K. *Ascospores* 8 per ascus, hyaline, simple, ellipsoid, 10–12 × 6–7 μm. *Pycnidia* punctiform, globose, immersed, simple with bluish-black ostioles, c. 260 μm diam., conidia hyaline, filiform, 10–25 ×  0.5 μm.

**Chemistry***.* Thallus K–, C–, KC–, PD–, medulla K–, C–, KC–, PD–. Secondary metabolites detected by TLC: isousnic ( +∕ −), usnic (+∕ −), psoromic (+∕ −) and fatty acids (+∕ −).

**Ecology and distribution**. On granite and volcanic rocks, in dry and sunny stations, at higher elevations (1,500–2,500 m) in North America.

**Comments**. *R. pseudomellea* is characterized mainly by its distinct rosette thallus with long, sinusoid, darker at the tips marginal lobes and orange-brown to reddish-brown, glossy upper surface. Compared to other species treated here, *R. pseudomellea* has a darker colour, longer and much less convex marginal lobes, as well as a distinctly areolate not squamulose thallus centre. However, its apothecia are usually very similar in colour and morphology to *R. phaedrophthalma*. *R. pseudomellea* has very variable secondary chemistry. The most often secondary metabolite occurring in the thallus is isousnic acid, but it may also contains psoromic acid.

**Specimens examined**. USA. CALIFORNIA: Kern Co., Eastern slope of the southern Sierra Nevada, BLM land on the divide between Oil Canyon and Pine Tree Canyon, along 4WD road which also serves as part of the Pacific Crest Trail, in a pinyon pine and scrub oak woodland, on white shale-like rocks, alt. c. 6150 ft, 19 Apr. 1997, *J.R. Shevock 15124* (ASU). OREGON: Harney Co., Steen Mountain (68 ml SSE of Burns), NW slope near snow patch, alt. 8700 ft, 18 July 2006, *C.M. Wetmore 95084, 95079* (MIN); Lake Co., Fremont National Forest, Along Dairy Creek, SE part of Gearhart Wilderness, alt. c. 1915 m, 27 Aug 1991, *B.D. Ryan 28456* (ASU); Lake Co., Fremont National Forest, Palisade Rocks, SE part of Gearhart Wilderness, c. 1950 m, 11 Sept. 1991, *B.D. Ryan 28652* (ASU); Tulare Co., Sequoia National Park, Milk Ranch Peak at border of park E of headquarters, around peak with white fir, incense cedar, pines and rocks, alt. 6100 ft, 21 May 1984, *C.M. Wetmore 51151* (MIN); Tuolumne Co., Winter Sports Area, N of Hwy 108, 5 km SW of Fraser Flat Campground, alt. 1700 m, on volcanic rock, pine, oak, 13 Aug. 1989, *B.D. Ryan 24402* (MIN).

## Discussion

*Rhizoplaca subdiscrepans* s. lat. was found to be highly polyphyletic by Leavitt et al. (2016), and as a result of multigene analyses, these authors delimited five cryptic species-level lineages within the species complex, defining them as *R. subdiscrepans* ‘subd A, B, C, D and E’. In parallel, another paper was published concerning the generic classification of lecanoroid lichens and including some representatives of the genus *Rhizoplaca* (Zhao et al., 2016). In the latter authors’ phylogenetic analyses, they also took into account several placodioid species previously classified in *Lecanora*, e.g., *L. opiniconensis* and *L. phaedrophthalma*. Among others, they included in their analysis single sequences of the two latter species, published by Arup & Grube (2000) but not treated by Leavitt et al. (2016). As a result, Zhao et al. (2016) found a core group of *Rhizoplaca* formed a monophyletic group together with the mentioned species and transferred these species along with some others to *Rhizoplaca.*

Both *R. opiniconensis* and *R. phaedrophthalma* were described as new taxa based on morphological and chemical features in the second half of the 20th century. However, it is presently known that morphological characters may not be sufficient for detecting taxa (Bickford et al., 2007; Crespo & Pérez-Ortega, 2009). On the other hand, careful morphological analysis of distinct phylogenetic lineages may lead to the recognition of some previously overlooked characters (Kroken & Taylor, 2001; Del Prado et al., 2007; McCune & Altermann, 2009; Frolov et al., 2016).

Species candidates within *R. subdiscrepans* s. lat. were considered to be cryptic by Leavitt et al. (2016), but in our morphological analysis, an attempt was made to identify potential diagnostic features for samples representing different clades. In contrast to recognized candidate species within the *R. melanophthalma* complex, which were highly variable (Leavitt et al., 2011), the morphology of *R. subdiscrepans* s. lat. representatives seem to be rather similar, especially in the case of clades ‘subd E’ (conspecific with *R. subdiscrepans* s. str.) and ‘subd D’ (conspecific with *R. opiniconensis*). The most common and characteristic features of all examined samples were a placodioid, polyphyllous, yellow-green thallus with squamulose-bullate centre, visible marginal lobes, and sessile apothecia with pale yellowish to brown, epruinose discs. However, despite their apparently similar morphology, the samples are heterogeneous and vary in the appearance of their apothecia and marginal lobes, as well as in the shape and size of ascospores and conidia. These characters have been shown to be diagnostic in the case of the Parmeliaceae family (Argüello et al., 2007; Divakar et al., 2010).

In our study, *R. opiniconensis* seems to be characteristic in its arched conidia and thallus colour that becomes distinctly more orange in herbarium material. Whereas the main characteristic feature of *R. phaedrophthalma* (conspecific with ‘subd A’) is the morphology of the apothecia, which are strongly convex with reddish-brown discs and an excluded thalline margin. Furthermore, some differences in the latter species are the size of the conidia, which are slightly larger than in the other discussed taxa, and ascospores that are distinctly smaller and subglobose rather than ellipsoid. Additionally, in contrast to other analysed representatives of *R. subdiscrepans* s. lat., thalli of mature specimens of *R. phaedrophthalma* are usually poorly placodioid, with weakly developed marginal lobes.

In the above morphological studies, we noted that none of the representatives of *R. subdiscrepans* s. lat. had orange and pruinose apothecial discs, which are mentioned in the literature as characteristic for *R. subdiscrepans* (Ryan, 2001). These characters, as well as the greyish-green tint of the upper surface of the thallus, are more appropriately applied to *R. chrysoleuca* s. lat. than to the *R. subdiscrepans* complex, an assumption that is consistent with the opinion of Cansaran et al. (2006). However, according to Zheng, Sheng & An (2007)*,* apothecial discs and their pruinosity do not indicate proper phylogenetic relationships among *Rhizoplaca* species, so this issue requires further research.

Chemical characters that are widely used for species delimitation in lichenology are not always good taxonomic indicators, especially when they do not correspond with molecular data (Hawksworth, 1976; Culberson, 1986; Ryan & Nash, 1997; Divakar et al., 2010; Thell et al., 2017; Ossowska et al., 2018; Zakeri et al., 2019). This fact may apply among others to the genus *Rhizoplaca* for which high chemical variability has been shown (Wei & Wei, 2005; Zhou et al., 2006; Leavitt et al., 2013a). On the other hand, in many cases chemistry may be successfully used as a character to support the circumscription of particular lichen taxa (Elix, Corush & Lumbsch, 2009; Leavitt, Johnson & St. Clair, 2011; Molina et al., 2011; Spribille, Klug & Mayrhofer, 2011; Onut-Brännström, Johannesson & Tibell, 2018; Mark et al., 2019). This was demonstrated within the *R. melanophthalma* species complex in the case of *R. parilis* S. Leavitt., Fernandez-Mendoza, Lumbsch, Sohrabi & L. St. Clair (Leavitt et al., 2013a).

With this in mind, we carefully analysed the content of secondary metabolites in the thalli of *R. subdiscrepans* s. lat. representatives sampled herein. The survey indicated such lichen substances as isousnic, usnic and placodiolic acids, as well as fatty acids and terpenoids, but some differences in their presence in particular recognized species could be observed. Among the secondary metabolites detected by TLC, neither isousnic acid nor fatty acids have been found in the thalli of *R. phaedrophthalma*, whereas the presence of fatty acids was observed only in specimens of *R. subdiscrepans* s. str., including those samples from Poland. None of the examined samples of *R. subdiscrepans* s. lat. contained psoromic, lecanoric or norstictic acids, as mentioned in the literature (Ryan, 2001).

*Rhizoplaca subdiscrepans* has until recently been considered to be distributed worldwide. However, phylogenetic analyses showed some slight differences in the geographical range of cryptic lineages within this species complex (Leavitt et al., 2016). All of the clades identified by Leavitt et al. (2016) included representatives on the Asian continent. Nonetheless, individuals belonging to clades ‘subd A’ and ‘subd D’ also occurred in North America, whereas clade ‘subd E’ had a European distribution. The results of our study correspond with theirs.

*Rhizoplaca opiniconensis* (= ‘subd D’) has been genetically confirmed as occurring in North America and is not found in Europe. The species epithet, however, is reported here for the first time from outside North America, i.e., from East-Central Asia (Altay). It should be noted that based on detailed habitat analysis of available samples it seems this taxon is hygrophytic, preferring mountain habitats in higher elevations, localized in shaded and moist places close to water resources. *Rhizoplaca phaedrophthalma,* same as *R. opiniconensis*, has not been noted from Europe, its centre of distribution being located in North America and Western and Central Asia, with the *locus classicus* in Nepal, which is in accord with the distribution pattern of clade ‘subd A’(Leavitt et al., 2016). The species prefers different habitat conditions than *R. opiniconensis*, since it occurs mostly on siliceous rock in semi-arid areas but also at higher elevations.

Finally, *R. subdiscrepans* s. str. (= ‘subd E’) besides Eastern Asia has been recorded in Europe (Poland and Ukraine) and has not been confirmed in North America. This is important in the light of the fact that *R. subdiscrepans* s. str*.* has been described from Switzerland in Europe. The distribution pattern of the species supports our concept that it is indeed conspecific with clade ‘subd E’ (Leavitt et al., 2016). *R. subdiscrepans* occupies specific habitats, occurring namely in warm, dry and sunny places in lower elevations, usually on volcanic rocks with a southern exposure. It is the only taxon of the discussed group of lichens with such ecological preferences. It is worth noting here that analysed samples of *R. subdiscrepans* s. str. are very similar in morphology to representatives of *R. opiniconensis*. Nevertheless, the species seems to be distinguishable based on its different geographical range and habitat preferences. These characters are often mentioned in literature as a supporting species recognition, even when there is a lack of evident phenotypic differences or they are insufficient to circumscribe the species (Crespo et al., 2002; Argüello et al., 2007; Divakar et al., 2010; Onut-Brännström, Johannesson & Tibell, 2018).

The above data can also be compared to the *R. melanophthalma* species complex presented in Leavitt et al. (2013b). Two of the identified phylogenetic lineages therein had intercontinental distributions, while four were found exclusively in North America. In this case, phylogenetic analyses, as well as geographical distribution, provided the basis for delimitation and description of new taxa (Leavitt et al., 2013a). In addition, *R. melanophthalma* s. str. was circumscribed and shown to be represented by a clade with the widest geographical range that also includes Europe.

During our study, a few sequences were generated for specimens representing *Lecanora pseudomellea* B.D. Ryan, a placodioid taxon occurring only in North America. The species forms a strongly supported monophyletic lineage within the *Rhizoplaca* and was therefore transferred to this genus. Characteristic features of this taxon are an orange-brown to reddish-brown, glossy upper surface, as well as long, sinusoid, darker at the tips, marginal lobes. Compared to other taxa discussed above, *R. pseudomellea* has a darker colour, longer and much less convex marginal lobes, as well as a distinctly areolate, not squamulose, thallus centre. It has a very variable secondary chemistry and the occurrence of compounds varies widely between samples. The most common substance occurring in the thallus is isousnic acid, but the species may also contain psoromic acid, which is not present in the other taxa of the discussed group.

## Conclusions

Despite the development of modern phylogenetic methods, species delimitation within lichenized fungi is still problematic. Implementation of integrative taxonomy and incorporation of various data, such as genetic, morphological, chemical, geographical and ecological data, usually deliver some resolution. An increasing number of analysed samples can also become more informative with these tools; these often lead to the conclusion that molecularly defined units are semi-cryptic rather than cryptic, as in the case of our study.

In our study based on molecular and phenotypic data, as well as in reference to previously described species, names are proposed for three lineages, the ‘subd A, D and E’ of *R. subdiscrepans* s. lat. delimited by Leavitt et al. (2016) and recognized respectively as *R. phaedrophthalma*, *R. opiniconensis* (supported by the placement of the type sequence in our phylogeny) and *R. subdiscrepans* s. str. Furthermore, we suggest transferring *Lecanora pseudomellea* to the genus *Rhizoplaca* with a proposal for a new combination—*Rhizoplaca pseudomellea*.

The geographical conclusion of our survey is that *R. subdiscrepans* s. str. appears to be mostly a European taxon with a range extended to Western Asia, whereas *R. opiniconensis* has a broader distribution than previously recorded, as it occurs not only in North America but also in Asia.

##  Supplemental Information

10.7717/peerj.9555/supp-1 Supplemental Information 1 New sequence data used in the study recently submitted in NCBI, not published

## Additional Information and Declarations

### DNA Deposition

The following information was supplied regarding the deposition of DNA sequences: The ITS sequences are available at GenBank: MN931719 to MN931739. The GenBank accession number, species, and location of the specimen (herbarium collection number) of each studied specimen are as follows:
MN931719 Protoparmeliopsis muralis, Szczepańska 1263 (hb. Szczepańska)
MN931720 Rhizoplaca opiniconensis, Brodo 25117 (CANL) Canadian Museum of Nature
MN931721 Rhizoplaca opiniconensis, Wetmore 88134 (MIN) University of Minnesota
MN931722 Rhizoplaca opiniconensis, Wetmore 74475 (MIN)
MN931728 Rhizoplaca phaedrophthalma, Ryan 31877 (ASU) Arizona State University
MN931729 Rhizoplaca phaedrophthalma, Ryan 31889 (ASU)
MN931730 Rhizoplaca phaedrophthalma, Ryan 32870 (ASU)
MN931732 Rhizoplaca phaedrophthalma, Wetmore 80571 (MIN)
MN931726 Rhizoplaca phaedrophthalma, Wetmore 80979 (MIN)
MN931723 Rhizoplaca phaedrophthalma, Wetmore 95059 (MIN)
MN931724 Rhizoplaca phaedrophthalma, Wetmore *5101* (MIN)
MN931731 Rhizoplaca phaedrophthalma, Wetmore 80866 (MIN)
MN931725 Rhizoplaca phaedrophthalma, Wetmore 81235 (MIN)
MN931727 Rhizoplaca phaedrophthalma, Wetmore 81446 (MIN)
MN931734 Rhizoplaca pseudomellea, Wetmore 51151 (MIN)
MN931735 Rhizoplaca pseudomellea, Ryan 24402 (MIN)
MN931736 Rhizoplaca pseudomellea, Wetmore 95079 (MIN)
MN931737 Rhizoplaca pseudomellea, Wetmore 95084 (MIN)
MN931733 Rhizoplaca pseudomellea, Ryan 28456 (ASU)
MN931738 Rhizoplaca subdiscrepans, Szczepańska 923 (KRAM)
MN931739 Rhizoplaca subdiscrepans, Szczepańska 967 (KRAM) W. Szafer Institute of Botany, Polish Academy of Sciences.

### Data Availability

The following information was supplied regarding data availability: The alignment of ITS sequences are available at TreeBASE: http://purl.org/phylo/treebase/phylows/study/TB2:S26365?x-access-code=cecc71c8fa6e3378ec581a812b7b976b&format=html.
